# Fatigue and quality of life of health professionals in Primary Care during the COVID-19 pandemic

**DOI:** 10.47626/1679-4435-2022-968

**Published:** 2024-08-05

**Authors:** Alberto Sumiya, Carla Fabiana Tenani, Carlos Podalirio Borges de Almeida, Juliberta Alves Macêdo, Eloisa Pavesi, Rafael de Menezes Reis, Julia Spengler, Fabiano Silva Locks Junior, Celita Salmaso Trelha

**Affiliations:** 1 Departamento de Ciências Morfológicas (MOR), Universidade Federal de Santa Catarina (UFSC), Curitibanos, SC, Brazil; 2 Faculdade de Odontologia, Universidade Estadual de Campinas, Campinas, SP, Brazil; 3 Instituto de Estudos em Saúde e Biológicas (IESB), Universidade Federal do Sul e Sudeste do Pará (UNIFESSPA), Marabá, PA, Brazil; 4 Instituto de Saúde e Biotecnologia (ISB), Universidade Federal do Amazonas (UFAM), Coari, AM, Brazil; 5 Departamento de Fisioterapia, Centro de Ciências da Saúde, Universidade Estadual de Londrina (UEL), Londrina, PR, Brazil

**Keywords:** primary health care, COVID-19, quality of life, fatigue, atenção primária à saúde, COVID-19, qualidade de vida, fadiga

## Abstract

**Introduction:**

The COVID-19 pandemic brought about an important discussion about the health of primary health care workers who are subject to physical and psychological distress, which may initially be expressed by fatigue and change in quality of life.

**Objectives:**

To verify the correlation between fatigue and quality of life of primary health care workers during the COVID-19 pandemic in Brazil inland.

**Methods:**

Cross-sectional, quantitative study, with the application of three questionnaires: social and demographic; Fatigue Perception Questionnaire; World Health Organization Quality of Life instrument-Abbreviated version. Statistical analysis comparing two or more groups and correlation adopting a significance level of p < 0.05.

**Results:**

It included 50 professionals with a mean age of 40.7 ± 9.6 years. High fatigue was evidenced (68.2 ± 17.2 points), and married individuals had a higher level of fatigue than single individuals (p = 0.003). There was also a high general average score in quality of life (85.27 ± 9.6 points), especially in workers with higher education (p = 0.03), as well as in non-smoking professionals (p = 0.02), with higher household income (p = 0.04) and in singles (p = 0.01). Therefore, the correlation was inverse and moderate between fatigue and quality of life (R = -0.44).

**Conclusions:**

We found a high level of fatigue and quality of life and an inverse correlation. The results show convergences and divergences with the scientific literature, indicating the need for more studies with primary health care workers.

## Abstract (pt)

**Introdução:**

A pandemia de covid-19 trouxe uma importante discussão sobre a saúde dos profissionais de saúde da Atenção Básica sujeitos ao sofrimento físico-psíquico, podendo ser inicialmente expresso por fadiga e alteração da qualidade de vida.

**Objetivos:**

Verificar a correlação entre fadiga e qualidade de vida de profissionais de saúde da Atenção Básica durante a pandemia de covid-19 no interior do Brasil.

**Métodos:**

Tratou-se de um estudo transversal e quantitativo, com aplicação de três questionários: sociodemográfico; o Questionário de Percepção de Fadiga; e o World Health Organization Quality of Life instrument-Abbreviated version. A análise estatística foi feita com comparação de dois ou mais grupos e correlação adotando nível de significância de p < 0,05.

**Resultados:**

Participaram 50 profissionais com idade média de 40,7±9,6 anos. Evidenciou-se fadiga elevada (68,2±17,2 pontos), sendo que indivíduos casados tiveram maior nível de fadiga que solteiros (p = 0,003). Evidenciou-se também alta pontuação média geral em qualidade de vida (85,27±9,6 pontos), principalmente em trabalhadores com ensino superior (p = 0,03), assim como em profissionais não fumantes (p = 0,02), com maior renda familiar (p = 0,04) e em solteiros (p = 0,01). Portanto, a correlação foi inversa e moderada entre fadiga e qualidade de vida (R = -0,44).

**Conclusões:**

Encontrou-se elevado índice de fadiga e qualidade de vida e correlação inversa. Os resultados mostram convergências e divergências com a literatura científica, indicando a necessidade de mais estudos com os profissionais de saúde da Atenção Básica.

## INTRODUCTION

In Brazil, primary health care (PHC) is the preferred gateway to the Unified Health System (SUS), which has ensured access to the health network. PHC workers take action on collective and individual aspects, solving frequent problems that are relevant to the health of communities in terms of surveillance, care, support, and continuity of treatment.^1^

Building on an understanding about the importance of PHC, the COVID-19 pandemic has reinforced the discussion about the need for social protection for health care personnel. The lack of investment in this dimension, coupled with unpredictable challenges and the disruption of personal and professional routines, has increased emotional suffering, physical exhaustion, and stigmatization,^2^ which are characteristics close to post-traumatic stress or secondary trauma.^3^

Among the most common health problems observed during the COVID-19 pandemic was the prevalence of anxiety and depression,^4^ in a scenario where physicians have been shown to be a highrisk group for suicide.^5^ Fatigue also appeared with a high prevalence in the 30 to 39 age group.^6^ In Brazil, specifically, there was a high rate of mental disorder diagnoses among nurses and nursing technicians.^7^

This means that there have been changes in quality of life (QoL) that go beyond health, encompassing the level of independence, social and family relationships, work, the environment, and even spirituality.^8^ In turn, fatigue can be one of the first signs of concern, when complaints are usually of weakness and exhaustion after minimal effort, with autonomic and depressive symptoms, muscle pain, dizziness, headaches, sleep disturbances, irritability, dyspepsia,^9^ muscle tension, and exaggerated alertness to pain symptoms,^6^ which implies reduced attention and performance and a higher incidence of errors.

The hypothesis of this study was that the COVID-19 pandemic favored the emergence of fatigue, affecting QoL. The rationale for conducting this study was the need to raise awareness of the repercussions of unhealthy work conditions on PHC. Thus, the objective was to verify the correlation between fatigue and QoL among PHC workers during the COVID-19 pandemic.

## METHODS

This is a cross-sectional, quantitative study with a sample of health care personnel working in PHC in the municipality of Curitibanos, SC, Brazil. We collected data between November 2020 and May 2021 in seven PHC facilities.

The inclusion criteria were working in PHC, being of either sex, and accepting to participate voluntarily and signing an informed consent form (ICF). The exclusion criteria were being on sick leave, maternity leave, or vacation. We used three questionnaires: a social and demographic questionnaire; an adapted version of the Perception of Fatigue questionnaire;^10^ and the World Health Organization Quality of Lifeshort version (WHOQOL-Bref).^11^

The Fatigue Perception Questionnaire had 30 Likert questions, ranging from “never” (1 point) to “always” (5 points), about drowsiness, concentration and attention difficulties, and the projection of fatigue onto the body. The sum of the scores for Perceived Fatigue ranges from 30 to 150, and the classification obtained is either low fatigue (30 to 62 points) or high fatigue (63 points or more).^10^

The WHOQOL-Bref, on the other hand, is a quick QoL assessment tool for the last 2 weeks.11 It consists of 26 Likert questions (1 to 5) divided into five domains: general (questions 1 and 2); physical (questions 3, 4, 10, 15, 16, 17 and 18); psychological (questions 5, 6, 7, 11, 19 and 26); social relations (questions 20, 21 and 22); and environmental (questions 8, 9, 12, 13, 14, 23, 24 and 25). As the questions in the dimensions are not grouped in a sequential numerical order, it is necessary to consult the questionnaire to find out the grade/variation.

The scores for each domain are transformed into means, which may or may not be turned into percentages, to interpret the results. The closer the score is to 100, the better the QoL.^8,12^ Please note that questions 3, 4, and 26 scores must be inverted for analysis.

The data was analyzed using GraphPad Prism v. 8.0. The normal distribution of the sample was checked using the Shapiro-Wilk test, the comparison between two or more groups using unpaired t-test and analysis of variance (ANOVA) using the Bonferroni post-test. Finally, the relationship between fatigue levels and QoL was analyzed using Pearson’s correlation test, with a p-value significance level of less than 0.05.

The research project was approved under opinion No. 4.361.276, of October 26, 2020.

## RESULTS

Fifty PHC workers participated in the study, mostly women (n = 45), with an average age of 40.7±9.6 years (Table 1). Overall, PHC workers had an average score of 68.2±17.2 points (high fatigue) on the Fatigue Perception Questionnaire. When comparing social, demographic, and economic data with fatigue, there was a statistically significant difference in marital status (p = 0.006): married people had a higher level of fatigue than single people (p = 0.003) (Table 2).

**Table 1:** Social and demographic characteristics

Variables	n (%)
Sex Female	45 (90)
Male	5 (10)
Education High school	31 (62)
Specialist	11 (22)
Undergraduate	7 (14)
Master’s degree	1 (2)
Occupation Community health worker	22 (44)
Nursing technician	11 (22)
Nurse	7 (14)
Dentist	2 (4)
Dental assistant	3 (6)
Nursing assistant	2 (4)
Physician	2 (4)
Dental technician	1 (2)
Working time (hours) 40	45 (90)
More than 40	2 (4)
20	1 (2)
8	1 (2)
Did not answer	1 (2)
Exercise No	34 (68)
Yes	16 (32)
Smoking No	42 (84)
Yes	7 (14)
Did not answer	1 (2)
Alcoholism No	34 (68)
Yes	15 (30)
Did not answer	1 (2)

**Table 2:** Comparison of social, demographic, and economic variables with fatigue levels

Variables	Mean	SD	p-value
Sex Female	62.75	17.88	0.51
Male	68.70	17.23	
Education High school	68.48	17.41	0.88
Undergraduate	67.72	17.27	
Occupation CHW	69.50	17.07	0.94
Assistants	68.20	10.23	
Practitioners	65.30	18.23	
Technicians	68.42	20.21	
Exercise Yes	64.47	14.48	0.31
No	69.94	18.24	
Smoking Yes	69.00	18.89	0.89
No	67.98	17.36	
Alcoholism Yes	65.87	16.02	0.54
No	69.19	18.11	
Marital status Married	82.38	10.31	0.01
Single	90.43	9.23	
Higher household income Yes	67.90	19.68	0.93
No	68.35	16.51	

CHW = community health worker; SD = standard deviation.

As a result, the overall mean WHOQOL-Bref score was 85.27±9.6 points, showing the following results in the following dimensions: general = 7.43±1.6; physical = 22.6±3.8; psychological = 21.1±3.4; social relations = 11.3±2.2; and environmental = 22.3±3.9. When QoL and social, demographic, and economic data were analyzed, personnel who had finished college had a better QoL than those who had only finished high school (p = 0.03), and those with a higher household income (p = 0.04) and who were single rather than married (p = 0.01) (Table 3).

**Table 3:** Comparison of social, demographic, and economic variables with quality of life

Variables	Mean	SD	p-value
Sex Female	91.00	10.00	0.23
Male	84.54	9.83	
Education High school	81.86	8.42	0.03
Undergraduate	89.47	12.01	
Occupation CHW	84.06	9.47	0.17
Assistants	77.00	8.18	
Practitioners	89.70	11.64	
Technicians	84.83	10.74	
Exercise Yes	88.00	13.50	0.24
No	83.26	8.66	
Smoking Yes	78.14	9.77	0.02
No	85.95	10.27	
Alcoholism Yes	85.47	9.98	0.78
No	84.52	11.04	
Marital status Married	82.38	10.31	0.01
Single	90.43	9.23	
Higher household income Yes	81.76	10.39	0.04
No	88.19	10.19	

CHW = community health worker; SD = standard deviation.

The analysis according to WHOQOL-Bref domains only found differences between marital status and the physical dimension (single: 24.7±11.3 vs. married: 24.4±11.3; p = 0.02) and marital status and the psychological dimension (single: 20.5±3.3 vs. married: 22.7±3.6; p = 0.02).

**Figure 1:**
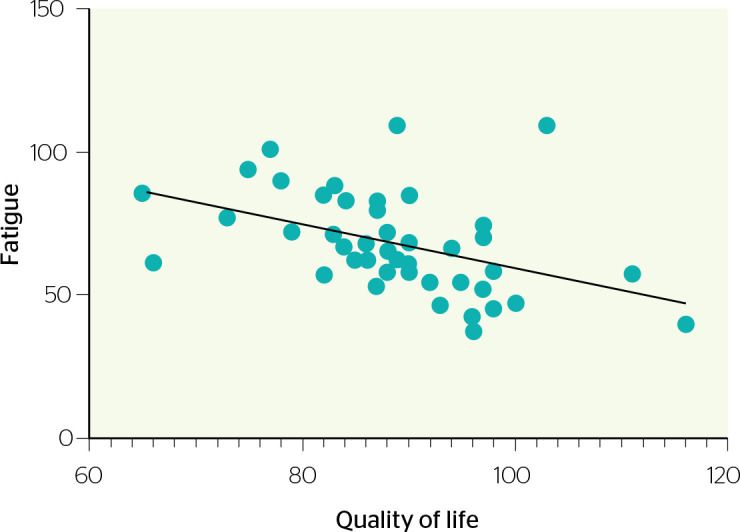
Inverse correlation between quality of life and fatigue level.

Finally, there was a moderate inverse correlation between the results of the questionnaires used to assess fatigue and QoL (R = -0.44, p = 0.003) (Figure 1).

## DISCUSSION

Literature has widely addressed PHC workers’ health conditions. However, it is common to emphasize the importance of prevention and precaution measures for workers’ health, not only to increase productivity, but also to increase recognition, satisfaction, and safety as factors that increase QoL.^13,14^

This study was conducted at a critical time of intense COVID-19 contamination in Brazil. In April 2020, 37,353 health care personnel had reported sick leave in Santa Catarina, including confirmed and presumed cases of COVID-19 contamination.^15^ As of March 2021, there had been 53 deaths from COVID-19, including 30 physicians and 23 nurses.^16,17^ The worst rates of stress were observed during lockdowns, as high infection and mortality curves demanded increased workloads.^4^ In other words, the domain of the workplace is the most compromised and determinant of QoL,^18^ which is further weakened with multiple employment relationships and the fear of contamination.^19^

In light of these circumstances, signs of fatigue are quite common and are often predictors of illness among professionals. These signs include apathy, irritability, a tendency towards depression with unspecific symptoms such as headache, dizziness, loss of appetite, insomnia, palpitations and tachypnea, feelings of guilt, reduced attention, clinical-therapeutic errors, absenteeism, and increased intention to terminate employment.^20,21^

Hence, this study found a predominance of women (90%), with a high school education (62%), working as community health workers (CHWs) (44%), working 40 hours per week (90%), and sedentary lifestyle (68%). The level of fatigue was classified as high, and there was a statistically significant difference in the marital status variable (p = 0.006), for which married individuals had a higher level of fatigue than single ones (p = 0.003). There were also high overall QoL scores, with statistically significant differences in education (p = 0.03), marital status (p = 0.01), smoking (p = 0.02), and higher household income (p = 0.04).

Although nurses seemed to be more affected in their QoL,^22^ it was found that CHWs were similarly affected when they had to modify/adapt their work routine in an emergency situation, exceeding the existing health needs. As a significant part of the frontline workforce, they were pushed to gain knowledge, improve practices, and use new tools and technologies to raise community awareness, engagement, and sensitization.^23^

A sedentary lifestyle is also an important wakeup call, as it is directly linked to a weakened immune system, which can potentially trigger or aggravate diseases. This clashed with the recommendations of social isolation and keeping up indoor exercise because for most people it was difficult or not implemented at all. A study on the COVID-19 pandemic and the level of exercise in adults revealed that age, the presence of chronic diseases, and a sedentary lifestyle prior to social distancing led to a higher risk of health impacts.^24^

This study found that unmarried workers had the best QoL. According to a survey of 306 health professionals in the state of Rio Grande do Sul, individuals who were married or had partners showed higher means in the social relationships domain, while the highest mean for widowers was in the environmental domain.^25^ On the other hand, the relationship between fatigue and education appears to be strong in lower educated individuals (unfinished elementary school/less than 8 years).^26^

Although most of the subjects in this study reported not smoking (84%), we found a statistical difference in relation to QoL. Smoking is seen as a negative influence and, it appeared as a coping behavior in the COVID-19 pandemic.^27^ Professionals with an income of less than two minimum wages scored poorly on the psychological and professional QoL questionnaire,^28^ as did those with more than one job.^19^

The COVID-19 pandemic has shown that protecting the health of health care personnel is fundamental to guaranteeing the functioning of the health care system and society, which is the opposite of what is observed in the SUS. In this respect, the World Health Organization points out some measures that should be considered: a) establishing synergies between policies and strategies for the safety of health care personnel and patients; b) developing and implementing national programs in favor of occupational health and safety for health care personnel; c) protecting health care personnel from violence in the workplace; d) improving mental health and psychological well-being; and e) protecting health care personnel from physical and biological hazards. In summary, it is understood that these measures also encompass the need for a development policy for human resources in health that values planning, regulation of working relationships, and continuing education for professionals and workers in this area, attention and self-care, and a reporting system for relevant cases.^29,30^

This study is limited to cross-sectional design, which prevents directly establishing a causal relationship. The sample was relatively small, but attention should be paid to the regional cut-off, which is relevant as more robust samples usually come from large centers or multicenter collaborations. The questionnaires used ensured a good assessment, though they did not cover all workplace and individual factors, such as differences in workflow between sites and the nuances of internal work or family relationships, which can have a direct or indirect effect.

## CONCLUSIONS

We found a high rate of fatigue and QoL, showing an inverse correlation. We recommend monitoring the variables analyzed with statistically significant differences, such as sedentary lifestyle, professional role and smoking, together with the most common signs of fatigue. Further studies on the subject are needed, as the information in the literature was both convergent and divergent with the results found.

In conclusion, the COVID-19 pandemic has led to the emergence, due to new occupational practices, of accusations and perceptions of vulnerabilities and risks to occupational health due to deteriorating working conditions as a result not only of insufficient investment but also of inadequate equipment, precarious labor relations and failure to provide incentives and/ or support for physical and emotional distress. The challenge is therefore to recognize the protection of employees’ well-being as an extremely important response to the rapid changes in the workplace.
